# Guide of the Practical Physician, or General Summary of Internal Pathology and Therapeutics, Clinically Applied

**Published:** 1844-10

**Authors:** 


### BRITISH AND FOREIGN
### MEDICAL REVIEW,

FOR OCTOBER, 1844.

### PART FIRST.

### Analytical and Critical Reviews.

### Art. I.

1. *Guide du Médecin Praticien, ou Résumé général de Pathologie Interne et de Thérapeutique appliquées.* Par F. L. J. Valleix, Médecin du Bureau central des Hôpitaux de Paris, etc. Tome I.—*Paris,* 1842.

*Guide of the Practical Physician, or General Summary of Internal Pathology and Therapeutics, clinically applied.* By F. L. J. Valleix, Physician to the Central Bureau of Hospitals of Paris. Vol. I.—*Paris,* 1842. 8vo, pp. 566.

2. *The Practice of Medicine; a Treatise on Special Pathology and Therapeutics.* By R. Dunglison, m.d., Professor of the Institutes of Medicine in Jefferson Medical College, Philadelphia, &c.—*Philadelphia,* 1844. *Second Edition.* Two Volumes, 8vo, pp. 632, 683.

The writer of one of the works whose titles stand at the head of this article, M. Valleix, in his well-written and well-reasoned preface, traces —and we think successfully—the causes which in France have produced a demand for works of a practical character. The physiological school of Broussais, which, though in the main false, had mingled with it enough of truth to give it an air of great plausibility, had obtained such entire possession of the minds of men, that tradition was interrupted, medical "history was become a dead letter," the yoke of authority was broken. But this medical revolution was shorter-lived than have proved certain political convulsions. Observation directed to the object of really illustrating the point in dispute—and it was so directed by that first of medical logicians, Louis—speedily demolished, even before its founder had quitted the scene, that ill-based fabric, the physiological system. But the traditional lore derived from antiquity had been extinguished by this system, whilst this had in its turn fallen before close observation and logical reasoning; and the followers of Broussais, in other words, almost the entire existing generation of French physicians, were at sea without a compass. Hence has arisen a demand for safe guides of practice, from an immense majority of practitioners of France.

From similar but not identical causes—for the doctrines of Broussais had but little influence on the British mind—the same demand exists in this country. In the early part of this century, especially after the publication of the works of Laennec, the current of the public mind set strongly towards pathological anatomy. Great expectations were entertained from it, and were, to some extent, realized in the improvement of diagnosis. But this result was not of a nature perfectly to satisfy the spirit—essentially utilitarian—of the profession in this country. They might for a time study medicine as an abstract science, but it would only be in the expectation of improvements in the *art* speedily resulting from it. But these results did not necessarily or speedily flow from pathological research. To recognize and name a disease was found to be one thing, to cure it another: the latter did not flow as a corollary from the former: it occurred as a contingency infinitely more rare than was expected, and disappointment was the result. A change came o'er the spirit of the age: "we want books useful at the bedside," was the cry; and, at once as an indication of the existence of this demand, and as a supply to meet it, the press poured forth 'Cyclopædias of Practical Medicine,' 'Libraries,' 'Dictionaries,' and treatises on the same subject, in rapid succession, from Craigie to Watson. Our transatlantic brethren abate nothing, as is well known, of the practical and utilitarian character of the Anglo-Saxon race whence they are descended, and were just as likely as ourselves to be soon weary of contemplating and classifying morbid products as one would objects of natural history, provided they led to no tolerably prompt result in the saving of life or the alleviation of suffering. With them, too, the demand is for therapeutics, and to meet this demand we have, (with others,) the work of Dr. Dunglison.

At first glance it might seem that whilst from M. Valleix we should derive a view of the condition of French practice exclusively, the work of our *quasi* fellow-countryman, Dr. Dunglison, would convey a knowledge of treatment as pursued solely among ourselves and our descendants. We do not find in either work, however, these exclusive views. Both are rich in references to opinions and practices prevalent among the best authorities in Great Britain and France. This is as it should be. An interchange of the treasures of mind, at least, constitutes a commerce in which there is an augmentation of general wealth, without impoverishment of any one. The general scope of the works is the same, the main object of both being therapeutics; both furnish so much of symptoms, causes, diagnosis, and pathological character as serves to render them intelligible guides to practice; and though their arrangement is not strictly identical, we can so adapt parts to each other as to furnish the reader with the opinions of both on any given disease at one view.

We select diseases of the respiratory organs as the ground on which first to show the characters of the works before us. Dr. Dunglison considers diseases of the larynx as the first in order in this department; not so, however, M. Valleix, who, prior to reaching the larynx, dedicates 180 pages to the classification, description, and treatment of diseases of the nasal cavities. We shall, however, commence at the lower point, and show how diseases of the larynx are handled by the respective authors.

Dr. Dunglison's classification of these diseases is exceedingly simple, viz. into acute, chronic, and œdematous laryngitis, designating by this last term what in this country and France is generally called œdema of the glottis—the name originally given to the disease by Bayle, who first described it, in which the sub-mucous cellular tissue of the larynx is occupied by a serous or sero-purulent infiltration. The division of M. Valleix is more complex, viz. into, 1st, simple acute laryngitis; 2d, simple chronic laryngitis; 3d, stridulous laryngitis, (pseudo-croup, laryngismus stridulus;) 4th, pseudo-membranous laryngitis, (croup;) 5th, œdematous laryngitis, (œdema of the glottis;) 7th, tumours of various sorts developed in the larynx; and, 8th, aphonia. It is evident from the respective classifications of the authors, that Dr. Dunglison has referred to a different compartment diseases referred by M. Valleix to the larynx. The former writer considers both croup and pseudo-croup (laryngismus stridulus) as laryngo-tracheal—correctly so, certainly, in the first case but with questionable accuracy in the latter.

Laryngitis. *Acute laryngitis* is the first disease of this class mentioned by both writers. In the account of this disease by M. Valleix we find more of system, more of the precise division of causes into predisposing and occasional, and more prolixity than in that of Dr. Dunglison; for though writers of our race are charged by a distinguished French author, Madame de Staël, with addiction to "longueurs," we cannot but opine that the charge of tediousness lies, with much more truth, against her own countrymen. French works, however, are so well put together, they are sent forth to the world so complete in all their parts, that we are led to pardon a somewhat undue consumption of time in their perusal, on the score of the workmanlike perfection and neatness of the manufacture. The symptoms of the acute and intense laryngitis of M. Valleix and of the merely acute of Dr. Dunglison nearly correspond. They are, pain in the larynx, never very considerable, increased in some degree by pressure on the upper edge of the thyroid cartilage. Deglutition is sometimes difficult; in some cases reported by English writers, says M. Valleix, liquids could not be swallowed. The same writer mentions the feeling as of a foreign body in the larynx as a symptom not less frequent than the preceding, and more distinctive. There is cough, attended, as the disease advances, with mucous expectoration—occasionally, Dr. Dunglison remarks, the sputa being tinged with blood. The voice is hoarse, but this hoarseness, at the expiration of a certain time, gives place to almost total extinction of voice, and, in a case reported by Dr. Wilson, (Medico-Chirurgical Transactions,) to its complete extinction. On auscultation of the larynx, says M. Valleix, there is a sonorous rhonchus or blowing sound (souffle), very intense and very harsh. M. Blaud has observed a very remarkable laryngo-tracheal mucous *râle*. Symptoms of asphyxia are naturally associated with the impeded passage of air through the larynx; hence the face is generally puffed and pale, the eyelids are swollen, and the lips blue, especially during spasmodic exacerbations, which are of frequent occurrence. Orthopnea, as might be supposed, generally exists. A comatose condition usually precedes dissolution. Fever, with a rapid pulse, accompanies the disease.

We annex from the work of M. Valleix a synoptical table of the diagnosis of laryngitis from affections with which it may be confounded :

| | |
|---|---|
| "*Laryngitis.* | *Pharyngeal Angina.* |
| "Pain on a level with the upper edge of the thyroid cartilage, increased by pressure ; deglutition of liquids little difficult. | Pain at the base of the lower jaw or the sides of the neck, augmented by pressure; swallowing very painful and difficult. |
| Voice hoarse, subsequently extinct. | Voice nasal and disagreeable. |
| Inspiration hissing; very great restraint of the respiration shortly after commencement of disease. | Inspiration simply painful; respiration is not restrained till a long time after the commencement of the disease. |
| Inspection displays only redness and swelling of the epiglottis. | Inspection displays swelling of the tonsils and the posterior part of the pharynx; the diagnosis is then very easy. |
| *Simple Laryngitis.* | *Croup.* |
| Expectoration of mucous, thready, feathery matters. | Expectoration of similar matters, but containing often fragments of laryngeal false membrane. |
| Inspiration as above. | Inspection often displays the presence of false membrane on the tonsils. |
| *Simple Laryngitis.* | *Acute ulcerated Laryngitis.* |
| Matters expectorated purely mucous. [Dr. Dunglison says they are sometimes bloody.—*Rev.*] | Streaks of blood in the matters expectorated. [A doubtful characteristic.—*Author.*] |
| *Simple Laryngitis.* | *Acute Œdema of the Glottis.* |
| Intervals of calm slightly marked. | Intervals of calm more marked. [A doubtful characteristic.—*Author.*] |
| Commencement of disease from a state of health. | Commencement in the course of an inflammatory affection, or of an acute ulceration in the vicinity of the larynx. |
| *Simple Laryngitis.* | *Foreign Bodies in the Larynx.* |
| Commencement gradual, though more or less rapid. | Commencement sudden. |
| Cough augmenting gradually in violence. | Sudden paroxysm of coughing, and at first of extreme violence. |
| Intervals of calm, as above. | Intervals of calm much more marked. |
| Fever from the commencement, and as much more intense as the local symptoms become more rapidly severe. | No fever at first, notwithstanding the severity of the local symptoms " |

We need scarcely remark that both our authors recommend vigorous antiphlogistic treatment for this affection when severe. Dr. Dunglison advises, as the first measure, the abstraction of blood from a vein, *till an impression is made on the system*, and the free application of leeches to the upper and front part of the neck. When the reaction which generally succeeds copious bloodletting takes place, he meets it by contra-stimulant doses of tartrate of antimony and potash, in doses ranging from one third of a grain to a grain, (anything under a grain we should consider a small dose in such a disease,) cathartics simple or cathartic enemata, and mucilaginous drinks. The patient should be forbidden to speak, and his chamber should be kept at a mild and agreeable temperature.

Should these not prove efficacious, a *revellent* treatment, he says, should be adopted, consisting of the free administration of mercury, so as to induce ptyalism. With this view, he recommends that either ten grains of blue-pill or one grain of calomel should be administered night and morning, whilst a drachm of mercurial ointment is rubbed into the thighs night and morning, and incisions made with the scarificator are dressed with the same ointment. Where spasms have intervened, and tracheotomy is not practicable, full doses of opium (three grains of soft opium in pill, or its equivalent in the preparations of opium or salts of morphia,) are indicated.

M. Valleix presents us his *methodus medendi* in a more detailed form than does Dr. Dunglison; but its general purport is, with an exception in the case of mercurials, very much the same as that of the latter gentleman. He arranges his plan of treatment in the form of prescriptions (ordonnances.) Of these the first for an adult is a light decoction of marshmallows and poppy-heads, edulcorated with gum-syrup; 2d, a bleeding from the arm, of twelve or fifteen ounces; 3d, fifteen or twenty leeches to the laryngeal region, to be repeated in the evening, if it appears necessary; 4th, vaporous fumigation, with the following decoction—marshmallows, about a drachm, and the head of a single poppy, boiled, in a quantity of water not specified, for twenty minutes. The vapour of this is to be directed during ten minutes towards the throat; the head to be covered carefully after each fumigation; the vessel containing the decoction not to be brought too near the mouth, in order not to augment the local heat: this process to be repeated night and morning. Two similar prescriptions, arranged for children aged from six to ten years, and for those from two to six years are then furnished, framed upon the same principle as the preceding.

M. Valleix admits, however, that the cases are rare in which we can limit ourselves to proceedings so simple; and that threatening suffocation soon compels the physician to resort to measures, if not more efficacious, certainly more energetic. Of internal means, he speaks favorably, for young children, of syrup of ipecacuanha, given in spoonfuls, till abundant vomiting is produced; for an adult, he recommends emetic tartar, in doses of one or two grains, combined with about a scruple of ipecacuanha, to be given in divided doses; or, he proposes, that two grains of emetic tartar should be dissolved in a pint of *tisan*, and that of this solution large drinks should be given, till vomiting and purging—which are productive of great relief—take place.

Under the head of external means we have every variety of rubefacient and blister mentioned: common blisters, ethereal extract of cantharides, frictions with croton oil, sinapisms, lotions of diluted muriatic acid—any of them to be applied near the part affected, or at some distance from it, as recommended by Drs. Arnold and Watson, (Med. Chir. Trans. Lond., vol. vi, p. 135, and vol. ix, p. 31;) but, excepting one, these various methods of accomplishing a rubefacient or vesicating purpose are all well known in this country. The one to which we allude M. Valleix praises, under the name of magistral blister (vésicatoire magistral), as a singularly prompt means of producing vesication; and we readily believe him. The following is the form: Take of powder of cantharides and of wheat flower each equal parts, and of vinegar a sufficient quantity to make a soft paste, to be applied to the skin. We think this form deserving of credit, both from the facility of its composition and, we feel convinced, for the promptitude and efficacy of its action.

We are subsequently presented (for the work is remarkable for the copiousness of its information) with a view of the practice adopted by different medical men in this disease. From this portion of the work we select the proceedings adopted by Dr. Henry Chavasse, in the case of a young girl, aged twenty, in the second day of the disease, with success. Blood was drawn from the arm, to the amount of forty ounces nearly, (1000 grammes;) eighteen leeches were applied to the neck; a purgative enema was given, and then a pill was administered every third hour, containing, (unless there be a very material typographical error,) fifty grains of calomel, and about one third of a grain of opium, along with a draught of liquid acetate of ammonia and camphor mixture.

These particulars will be found at p. 228 of the first volume of the work of M. Valleix. That a gross error exists there can be little doubt, at once from the extravagance of the dose of calomel, and the utter disproportion between the mercurial salt and the opium, although the table of *errata* throws no light upon it. We are sorry to observe such faults in a work destined for students of all ages, for the inexperienced may be led by them into errors actually fatal, whilst into those more advanced they are calculated to infuse distrust of a (generally) very valuable book. We think such faults very likely to occur, as the table of *errata* (though it has by no means comprehended all it might,) proves, from what we consider the very inconvenient mode adopted by the French, of stating all quantities in "*grammes*," or fractions of a "gramme," as a consequence of which writers are thrown, in expressing many quantities, into the decimal form, in which errors are very apt to occur. To take the example before us, the prescription runs thus:

| | | |
|---|---|---|
| ℞ | Calomel . . . | 16 grammes. |
| | Opium, in powder . . | 0·1 gramme. |
| | Conserve of roses . | q. s. |

Make six pills.

Now we feel no doubt that the first line of the prescription should be written, "calomel 1·6 grammes;" in fact, that the printer, as he might easily do, has multiplied the dose of Dr. Chavasse by ten.

Even with this obvious and necessary correction, the practice of Dr. Chavasse, in the bleeding and mercury alike, is bold and decisive; but probably not more so than the circumstances of the case before him demanded, he having been called on the second day of a very severe disease. We do not hesitate to admit that it is more to our taste than the more moderate proceedings which win the favour of M. Valleix. For our own part, we never can treat acute laryngitis without having the narrowness of the chink of the glottis before our eyes. The treatment of Dr. Chavasse is not, however, the object of especial censure from M. Valleix; the former writer, nevertheless, must accept his share of the following general denunciation:

"Calomel invariably constitutes part of the treatment employed by English physicians in acute laryngitis, and they all concur in praising it very much; but we should seek in vain in the facts for a confirmation of what they advance. In all cases, in fact, the calomel is associated with active means, such as blisters and even tracheotomy; and it is impossible to distinguish not only what is its effect, but even whether it have an effect. We shall see in our exposition of the practice of Dr. Chavasse, how calomel is administered." (Valleix, t. i, p. 222.)

Had M. Valleix employed a treatment absolutely simple, had he relied on bloodletting alone, he might, perhaps, without presumption, have cavilled at the more complex proceedings of others. But, considering the nature of his practice, may we not with as much reason as he can plead, say, " Decoction of marshmallows and the head of a poppy form invariably part of the treatment recommended by M. Valleix, but being as invariably associated with other means, how do we know what is its effect, or whether it has an effect ?" In a similar strain may we proceed with his fumigations, his ipecacuanha, his blisters, his emetic-tartar, and even with his bleeding—what is their effect, or have they an effect? M. Valleix seems to forget that medicine—if a science at all—is a science of observation ; experiment has led some one to the conclusion that mercury does good in inflammatory diseases, laryngitis included ; the information spreads, experiments are multiplied, and the universality of its employment by British practitioners—which M. Valleix mentions apparently as a censure—is evidence of the successful issue of many of these experiments. The experiment, too, of Dr. Chavasse was successful, which is more than M. Valleix ventures to predicate of his own more timid proceedings ; the only case he mentions, besides that of Dr. Chavasse, which resulted in recovery, being one by Zacutus Lusitanus, who bled a pregnant woman seven times in one day on account of laryngitis, of which she was cured.

Other means failing to give relief, tracheotomy must be resorted to, says Dr. Dunglison ; adding, however, and with truth, that it is of no use unless performed early, and before the patient is evidently sinking. Ryland has collected from various quarters twenty-eight cases of the disease ; in six of these the operation was performed, and in four a cure was effected ; in the other two temporary relief only resulted. The saving of the lives of two thirds, however, is a strong incentive to the operation. The operation, M. Trousseau says, frequently fails, from too small a canula being employed. He recommends a middle-sized canula at first, and gradually increases the size, until the air almost ceases to make a noise in passing through it during a deep inspiration.

M. Valleix does not appear to advocate *early* tracheotomy, as does Dr. Dunglison. He says that modern physicians are agreed that when suffocation is *imminent* the operation is indicated. Our own experience tells us that this may prove too late; for the last case we saw in which the operation was attempted, was under such circumstances, and the patient expired before it was completed. It is true that this operation was rendered tedious by the almost ossific rigidity of the rings of the trachea; but what we saw of the case convinced us that, without this untoward obstacle, the issue would have been the same. It appears to us, that, by delaying the operation till near the point of suffocation, we incur the risk indicated by Riverius, in words quoted by M. Valleix, of having the death of the patient imputed to the medical attendants: *propter metum infamiæ, quæ medicis et chirurgis impendet, dum æger post operatione occumbit;* and, what is much worse, of having deserved it.

*Chronic laryngitis.* The chronic form of laryngitis receives, as it merits, an ample share of the attention of both our authors. There is a copious reference to authorities in both of them, indicating an appeal to the same quarters by both. The names of Ryland, Andral, Louis, Graves and Stokes figure equally in the pages of the American as of the Gallic writer. The latter, with that fondness for method which characterizes him as an author, divides the disease into the slight (légère) and intense forms. We think the simpler plan of Dr. Dunglison — who gives the symptoms, with an intimation that cases vary in intensity—the more elligible one.

We copy the symptoms, as detailed by Dr. Dunglison :

" Pain is felt in the larynx, but its precise situation may vary; at times it extends over the larynx, but at others is restricted to a small space, and generally to the region of the thyroid cartilage. Commonly a kind of tickling sensation exists, which provokes coughing. The pain, too, is exasperated by coughing, speaking and deglutition, especially when ulcerations exist, and they are situate above the ventricles of the larynx.

"The breathing of cold air, and pressure upon the larynx likewise augment it. The voice is almost always changed, being hoarse and, at times, so much enfeebled as to be inaudible. The aphonia may supervene suddenly or gradually and ultimately be complete.

" Cough is a constant concomitant; and when the mucous membrane is much swollen it becomes hoarse and even croupy. In the first instance it is dry, but subsequently it is accompanied with the expectoration of mucus, mixed occasionally with pus or blood. At other times a membraniform matter is expectorated for months, and at others a considerable quantity of false membrane is thrown off, after which the patient rapidly recovers.

" Occasionally portions of cartilage are mixed with the mucus, or bloody sputa, and, in such cases, there is always accompanying hectic. Chronic laryngitis has indeed been divided into two heads: the first comprising that which affects the mucous membrane and the submucous tissue, and the second that which implicates the cartilages; the latter, it has been conceived, having perhaps the best claim to the name phthisis laryngea, from the incurable nature of the affection, and the hectic and emaciation which invariably accompany its latter stages. (Ryland.)

" When chronic laryngitis is slight, and there is not much narrowness, the difficulty of breathing may not be great; but if it be attended with much tumefaction of the lining membrane the dyspnea is considerable, and the sound rendered, on inspiration, sonorous and peculiar. It is evidently, too, augmented by paroxysms. The air of inspiration likewise gives rise to a snoring (ronfflement), or whistling (sifflement), which may be continuous, or recur in paroxysms. These local symptoms may be so slight that the general health does not suffer to any great degree. Commonly, however, more or less sympathetic febrile disorder is apparent, under which nutrition falls off, and atrophy supervenes. The disease now merits the term 'laryngeal phthisis,' which is, however, in the immense majority of cases, connected with the presence of pulmonary tubercles. (Andral.)

" Chronic laryngitis may be primary, or it may succeed to acute laryngitis; and when apparently terminating in health, it is readily reproduced by exposure to cold, errors in diet, &c. &c. Its duration varies from a few months to several years. When the fauces are inspected, but little evidence of disease may be perceptible; at other times, however, the mucous membrane is injected, and the follicles are so large as to resemble split peas. Whether this enlargement of the follicles is the cause or effect, may admit of a question. The enlarged follicles probably exist lower down, where they cannot be inspected. This form of laryngitis is the one often known under the name of " clergyman's sore-throat." Chronic laryngitis may terminate in health, but it is more likely to end fatally; and this may occur in different modes, either by the lungs becoming implicated, or by the extent of the laryngeal lesions themselves, which may excite severe irritative fever, or interfere with the entrance of air into the lungs, and thus induce asphyxia. In almost all cases of phthisis laryngea the disease is complicated with pulmonary tubercle. (Andral, Louis, Stokes.)

"Dr. Stokes, indeed, asserts that, after ten years of hospital and private practice, he never saw a case presenting the symptoms of laryngeal cough, purulent or muco-purulent expectoration, semi-stridulous breathing, hoarseness or aphonia, hectic, and emaciation, in which the patient did not die with cavities in his lungs. In some the laryngeal affection appears to be primary; but, in the great majority, symptoms of pulmonary disease existed previous to its appearance. Such also is the result of the author's observation. In many cases of pulmonary phthisis, sore-throat, hoarseness, or aphonia, with cough occur; but the case is different when the laryngeal symptoms have been primary. (Graves.)"—(Dunglison, pp. 235-7.)

We cannot help thinking that Dr. Dunglison has comprehended at least two different diseases in this description—what has been called (not very correctly) laryngeal phthisis and chronic laryngitis. We concur in the view of Dr. Stokes that those who manifest the symptoms of the former disease die with cavities in the lungs; but our experience does not lead us to the conclusion that the laryngeal affection is ever the primary condition in phthisis, as this able writer supposes it to be in a small proportion of cases. In all cases of what has been supposed to be laryngeal phthisis we have found on our first examination indubitable signs of cavities or other disease of the lungs already existing, so that our opinion would at the least accord with that of Andral, that "it is probable that idiopathic chronic laryngitis *rarely* [we should say *never*] produces the symptoms of phthisis; but the two diseases are frequent concomitants." In this latter clause we unequivocally concur; indeed the observations of the accurate Louis have shown that in one fourth of the cases of phthisis ulceration of the larynx has been observed; in one sixth ulceration of the epiglottis, and ulceration of the trachea was met with more frequently than either of the other lesions.

Now we cannot help thinking that to treat of such a condition as this, which is evidently part and parcel of phthisis, under the name of chronic laryngitis, and as an independent disease, is a gross violation of nosological precision, and, what is more, is calculated to lead to results practically pernicious. On this head M. Valleix expresses himself with much good sense:

"Non-syphilitic and non-cancerous ulcerated laryngitis is *almost* (?) always a complication of phthisis pulmonalis. This is a point which we shall subsequently discuss. So that whatsoever enables us to recognize incipient phthisis (for in the case of confirmed phthisis there is no difficulty,) will serve to establish the diagnosis. Emaciation, cough, night-sweats, feebleness, and, above all, alteration of the sound of the thorax and of the respiratory murmur under the clavicles, ought to be carefully investigated, for none of these symptoms exist in simple idiopathic laryngitis. In some cases, the examination of the patient not having been made with due care, laryngitis alone has been supposed to exist, though this has not been the case: this proves that the diagnosis is sometimes very difficult, for the pectoral symptoms, which alone can guide us, are then very trifling." (Valleix, t. i, pp. 242-3.)

Dr. Dunglison's prognosis is rendered exceedingly and very unnecessarily gloomy by the confusion—he must pardon the expression—which appears to pervade his mind regarding the nature of the disease. When he says "chronic laryngitis may terminate in health, but is more likely to end fatally," he must be understood as expressing, in the first clause of the sentence, what is probable, and highly probable, with respect to the disease, which ought alone to have been considered on the present occasion; in the second he must be regarded as stating the truism, that phthisis is generally fatal. The prognosis of M. Valleix is of a very different description. He says:

"According to those authors who have paid most attention to chronic inflammation of the larynx, simple laryngitis tends naturally to a cure; the excesses and imprudences of the patient alone keep up the disease and often aggravate it." (Valleix, t. i, p. 246.)

There is, however, a chronic laryngitis existing as an independent disease; and this is clearly exemplified in what Dr. Dunglison calls, and by no means inappropriately, the "clergymen's sore-throat." It is certainly not limited to gentlemen of the clerical profession; but they being more exposed than most classes of the community to one of its causes, inordinate exertion of voice, it prevails very much among them, especially among the younger and unseasoned portion. It should be remarked, however, that men whose vocation is not speaking would neglect an affection of the larynx, for which a clergyman, who is called upon once if not twice a week to address a large congregation, would hurry to his medical adviser for assistance:

"For he who lives to preach, must preach to live."

Hence it would not be correct to consider that it prevails among clergymen in comparison with the rest of the community, in the proportion in which the former class seek medical relief from it.

There is sometimes in this disease, for there is but one simple chronic laryngitis, whether it affects clergymen or others, injection of the mucous lining of the fauces, and enlargement of the follicles; whilst on the other hand these conditions are frequently not perceived, but the laryngeal affection is clearly denoted by very slight pain, increased by inspiring cold air, pressure and exertions of voice, when it becomes such as to compel the patient to cease speaking. The sensation frequently does not amount to pain, only to a very unpleasant tickling, but equally with pain exciting cough, and compelling the patient, if speaking, to stop instantly. The condition of the voice often varies in the same patient in the same day, being for a period perhaps little modified, at other times very hoarse, then muffled and very feeble so as to constitute almost total aphonia. There is sometimes no constitutional disturbance in the disease, and when any exists it is very slight. Simple chronic laryngitis is a common sequel of influenza.

Besides simple chronic laryngitis, and that form of affection of the larynx which is associated with phthisis, this organ may be affected with syphilitic and cancerous ulceration. There will not be any considerable difficulty in discriminating between these diseases and the simple affection now under consideration. The diagnosis of M. Valleix seems to us succinct and precise. The symptoms of cancerous laryngeal affection, he says, are emaciation, feebleness, languor; pale yellow complexion (cancerous cachexia); severe pain; crepitation sometimes produced by pressure on the cartilages of the larynx; expectoration of muco-purulent matters and cartilaginous *detritus;* and sometimes thickening, deformity, and ulcers of the epiglottis. Syphilitic disease of the larynx is to be distinguished by antecedent syphilitic infection; venereal ulcers, and eruptions in other parts of the body, exostoses, &c.; at an advanced period expectoration of muco-purulent matters, and syphilitic cachexia. There is, as a general rule, not much difficulty in discriminating syphilitic affections of the larynx from simple laryngitis or from phthisical disease of the organ. A case, however, in which we witnessed the confusion of the syphilitic with the phthisical form is fresh in our memory; but a syphilitic ulcer on the posterior pillar of the palate, the lower portion of which could be traced to the side of the larynx, where it was lost, and a little confidential conversation with the patient decided the case in our mind; whilst a moderate mercurial course decided it in the opinion of the family, for a patient supposed to be consumptive was in a month restored to perfect health.

The remedial measures suggested by both our authors may be stated to be those, in a mitigated and more continued form, recommended by them for the acute disease. Both mention bleeding from the arm as advisable in some cases at the commencement of the disease; leeches to the laryngeal region are suggested; and M. Valleix, on the authority of MM. Trousseau and Bellocq, cupping the nape. Counter-irritants (revellents) are praised by both; and the whole tribe of these remedies—blisters, ointment of emetic tartar, frictions of croton-oil, the application of caustic potash every eight days on the side of the larynx (recommended by MM. Trousseau and Bellocq), and a small seton under the skin on each side of the neck near the larynx (as suggested by Dr. Baillie), are passed in review. We need scarcely remark that tisans form part of the measures of M. Valleix. He recommends those of rather a stimulant or tonic character, and names a decoction of bardana sweetened with syrup of fumitory, or one of ground-ivy adulterated with syrup of capillaire. Medicines of the narcotic class form an important part of the prescriptions of both our authors. Laudanum and tincture of digitalis in conjunction are recommended, by Dr. Dunglison, to be given internally in doses of about six minims each three times a-day; and the same authority suggests the endermic employment of the salts of morphia, half a grain of the acetate or sulphate being sprinkled over a blistered surface. M. Valleix, from the same class, besides opium and lactucarium, the authority for which quoted by him is Dr. Walker, (in the London Med. Repository for 1822,) suggests the use of extract of hemlock, as prescribed by Dr. Baillie. These are a part of the many evidences furnished by his book of the really very extensive acquaintance of M. Valleix with British medical literature. The inhalation of narcotic vapours is recommended, especially by M. Valleix. The form he suggests is, infusion of elder-flowers about three pints, powder of stramonium a drachm nearly; the vapour to be inhaled, for which purpose a common teapot answers very well; or the same medicine may be smoked in a pipe, as prescribed by M. Cruveilhier. For this purpose, a scruple (nearly) of the dried leaves of the datura stramonium and an equal quantity of the leaves of sage should be used for a pipe, and this quantity may be smoked daily or even three times a day. The leaves of belladonna may be substituted for those of stramonium; but in this case the dose should be increased by about a fourth or fifth.

Topical applications directly to the membrane affected, either in a liquid state or in powder, are mentioned by both our authors. Dr.

Dunglison prefers them in solution, and regards the nitrate of silver as the best of the class. It may be used in the proportion of ten or fifteen grains to the ounce of water, as advised by Graves and Stokes, and it has been prescribed by MM. Trousseau and Bellocq as strong as one part of the nitrate to two of water. The plan suggested by Mr. Cusack, of Dublin, for its application he considers as the best. A brush of lint of the requisite size is sewed on the end of the finger of a glove, which is then drawn on the index-finger of the right hand. The patient is made to gargle with warm water, and the lint being dipped in the solution can be readily applied to the larynx.

The acid nitrate of mercury has been recommended for the same purpose as the solution of nitrate of silver; but M. Valleix cautions us against it on two grounds,—the danger attending its use, and the want of positive information as to any favorable result hitherto obtained from it. Dry powders have been recommended to be drawn or blown into the larynx. The powders, according to M. Valleix, used for the purpose are, 1st, sugar; 2d, subnitrate of bismuth, either pure or mixed with an equal weight of sugar; 3d, sulphate of zinc 1 grain mixed with 36 grains of sugar; 4th, sulphate of copper and sugar in the same proportions as the sulphate of zinc; 5th, alum, 5 grains mixed with 10 of sugar; 6th, acetate of lead, about 2 grains mixed with 14 of sugar; 7th, nitrate of silver, 1 grain mixed with 72 of sugar. M. Bretonneau has suggested a cumbrous apparatus for introducing these powders into the larynx; but the purpose seems better answered by an open glass tube, or, what was used by Aretæus in angina maligna for a similar purpose, a reed. Into one or the other of these instruments the powder is introduced, and the instrument is placed in the mouth; and by a sudden inspiration after a complete expiration, the powder is conveyed into the throat. A portion rests on the pharynx; another portion is conveyed into the larynx, and the patient is cautioned to repress coughing that it may rest the longer in contact with the laryngeal lining. The testimony in favour of this mode of medication is not so strong as for liquid cauterization with nitrate of silver.

Mineral waters taken at the spring are recommended by M. Valleix, on the authority of MM. Trousseau and Bellocq, and Louis. The waters recommended are the "Eaux-Bonnes," those of Cauterets, of Saint-Sauveur, and of Luchon. We have seen ourselves the greatest benefit from travelling, especially from a cold and damp to a warm and dry climate. The effect is not suddenly brought about, but we have seldom found it fail ultimately to effect a cure.

*Œdematous laryngitis.* That very distressing and dangerous disease, œdema of the glottis, is discussed by M. Valleix under the title of œdematous and sero-purulent laryngitis, whilst Dr. Dunglison simplifies the name by omitting the latter adjective. Both writers take much pains to indicate the diagnostic marks between this disease and acute laryngitis. Both are agreed that in the early stage of *œdema glottidis* its symptoms are simply those of a mild degree of the former disease. As the disease advances, the diagnosis becomes more clear. M. Valleix arranges the diagnostic marks in a tabular form. They are in substance as follows. In *œdema glottidis,* there are very violent paroxysms of dyspnea, in acute laryngitis the paroxysms are less marked and violent; in the former disease, inspiration is singularly sonorous, sibilant, and difficult, expiration being comparatively easy and exempt from sound, whilst in the latter, respiration is not marked by any peculiar noises, nor is there that discrepancy between its two parts, which is manifest in the former; in œdema of the glottis, the quantity of air entering the lungs being very small, the respiratory murmur is very feeble indeed, almost abolished throughout the thorax, whereas in simple acute laryngitis, this murmur is rather loud. Finally, in œdema, we can by carrying the finger to the upper part of the larynx, detect there a soft ring or cushion, caused by the infiltration of serous or sero-purulent fluid into the submucous cellular tissue, which is of course wanting in laryngitis.

The affection of the breathing is exclusive of the swelling round the larynx, one of the most distinctive symptoms of the disease. According to M. Valleix, inspiration is extremely difficult, requiring the greatest efforts on the part of the patient, and amounting frequently to true orthopnea. On the contrary, expiration takes place with so much ease, that M. Blache regards it as a pathognomonic sign. During inspiration there is often produced a wheezing, a guttural snoring, often very loud. One observer, M. Legroux, has remarked a peculiar sound like the crowing of a cock, or the sudden closing of a valve at the conclusion of each inspiration. Another indication of the great restraint of inspiration is the necessity which patients experience of keeping the mouth half open, notwithstanding which, the *alæ nasi* are expanded to the utmost at each inspiration.

This disease is generally fatal, Bayle having observed but one recovery out of seventeen cases. What is to be done to avert a fatal result? The pulse being often moderate, and there being seldom much heat of skin, antiphlogistic remedies are seldom required to the same extent as in laryngitis; yet leeches and blisters are generally advisable. Antimonial emetics afford relief, and with this view the potassio-tartrate of antimony may be given every two hours, in doses of two or three grains. Mercurial inunction is sometimes associated with the employment of the antimonial solution; or, as Ryland suggests, instead of the antimonial solution, five or ten grains of calomel are given every hour, with a view either from their purgative action, or from their revellent effect on the general system, of checking the inflammation of the glottis, and promoting the absorption of the effused fluids.

"Should these remedies," says Dr. Duglison, "have been employed, and attacks of impending suffocation be urgent, the operation of bronchotomy must be practised; but, as in cases of ordinary laryngitis, it must not be postponed too long, otherwise it also must fail. It has been proposed, that, the moment the nature of the disease is known, the operation should be practised, and that it should be followed up by the revellent treatment just recommended; (Vavasseur;) or that in its place, and the plan is preferred by some, an elastic gum-tube, open at both extremities, and of a proper size, should be introduced into the larynx from the mouth, so as to keep the passage open, (Bayle, Thuillier.) It is scarcely possible, however, to conceive that any benefit could acrue from the presence of an extraneous body in such a condition of the parietes of the larynx. [We should add, too, the difficulty of conceiving that such a body could for a moment be borne—Rev.] Another plan, scarcely, if at all, more feasible, is to pass the finger into the glottis, and to make pressure, so as to diminish their volume, (Thuillier;) and lastly, it has been proposed to scarify the engorged parts, (Lisfranc,) so as to permit the escape of the fluid. Both these plans have been characterized as fantastic, very difficult, if not impossible, of accomplishment, and more likely to increase than diminish the existing evil. (Ryland.) The last is undoubtedly extremely difficult of execution; but, if it could be carried satisfactorily into effect, it would offer great probability of benefit. The danger of the disease is, in some measure, proportioned to the extent of the tumefactions, and any method that would abstract the effused fluid could not fail to afford essential relief." (Dunglison, p. 242.)

It should be remarked that M. Lisfranc, (Journal gén. de Méd. t. lxxxiii, 1823,) who had the merit of first suggesting the scarification of the engorged parts, quotes five cases in which this operation was followed by immediate relief, and ultimately by a complete cure. In a sixth case many similar operations, performed at variable intervals, served only to palliate. There were deep lesions of the larynx, which ultimately destroyed the patient.

*Stridulous laryngitis.* We find ourselves very much puzzled to determine whether M. Valleix means by stridulous laryngitis, (laryngite striduleuse, pseudo-croup,) a totally different disease from the laryngismus stridulus of Dr. Ley, or whether he is taking a different pathological view of the same disease. He considers his stridulous laryngitis as a disease recurring in severe paroxysms with long intervals, during which there is very slight, if any, indisposition.

His description of the paroxysm is as follows:

"At the same time, the breathing is rapid, interrupted, and panting, and there is produced during inspiration an acute sound, more stridulous, more broken than that of hooping-cough, and which has been described by the names of stridulous, hissing, hoarse, sonorous respiration, and the crowing of a cock. It is especially the English physicians who have employed this last expression. These phenomena deserve all our attention, because for a long time they served to characterize croup; whereas it results from recent observations, that they belong rather to stridulous laryngitis than to croup properly so called. It is these peculiar cries which have procured for the disease the names of crowing inspiration, stridulous laryngitis, &c.

"When the child experiences a little relief, he complains or he cries, and then we can observe the modifications of the voice. It is hoarse and broken, yet always preserves a certain degree of force. I find in the writings of English authors, some cases in which *aphonia* is mentioned; but the subjects in which this symptom has been observed, were not attacked with a simple stridulous laryngitis; they experienced nervous attacks of which the suffocation and aphonia were a mere complication; sometimes, however, the voice may be extinct during the paroxysms. Jurine and M. Bretonneau have mentioned instances of it, but they are rare." (pp. 282, 283.)

"Whilst the infant experiences these distressing symptoms, he labours under great anxiety. His face is congested; his lips, especially, become violet; a little later, a frightful paleness is observed, and his eyes express terror. Dr. John Henderson (Observations on the Diseases prevalent among the Natives of Hindostan) has observed that the eyes are haggard. The patient throws his head backwards to breathe; he carries his hand to his neck; *there is sometimes a degree of distortion of his limbs, or feeble convulsions during the efforts of inspiration;* we discover, in one word, all the signs of asphyxia. Such is a paroxysm of stridulous laryngitis in its highest degree of intensity." (p. 284.)

The evidence adduced by M. Valleix for regarding this disease as a slight inflammation of the larynx is of the feeblest kind. He says that authors are agreed that there is little or no fever in the affection described by him; and in fatal cases pathological lesions in the larynx are very slight or null, "a little mucus, a feeble redness, have been observed in some cases, and, most frequently, authors have been content with saying that the larynx is exempt from change." He speaks of the lesions which some English authors have found in the membranes of the brain as indicative of a totally different disease from the one he is treating of, saying that in this case the principal malady was an arachnitis, of which the suffocation was only a symptom. "Dr. Clarke," he says, "attributes the disease to cerebral irritation; but it is a malady foreign to the larynx, *eclampsia*, which has led him into error." He speaks slightingly of the opinion of Dr. Ley, who attributes *laryngismus stridulus* "to tumefaction of the bronchial glands, which, compressing the *nervus vagus*, determine a mortal spasm of the glottis." But how slight soever the evidence in favour of his own view, he is not the less disposed to consider the question as definitively decided in his favour, and all opinion at variance with his own is stigmatized as error, as in the following passage:

"I have pointed out some slight convulsive moments at the height of the paroxysm, and at the moment when the suffocation is most alarming. Nevertheless many English physicians, among whom I shall cite principally Henry Rees, (On a Species of Convulsions in Infants, etc. in the Lancet, vol. i, p. 679, 1831,) J. Cheyne, (the Cyclop. of Pract. Med., art. Croup,) Clarke, (A peculiar Species of Convulsion in Infant Children,) have described at length convulsions which take place in some children, and have insisted much on their importance. The perusal of their cases and of their general descriptions, have convinced me that they have regarded as belonging to stridulous laryngitis the real convulsions of eclampsia, whether a stridulous laryngitis had complicated this last disease, or that the spasm of the glottis had been in these cases but a simple symptom." (Vol. i, p. 285.)

Again he remarks:

"The two most accurate observers, MM. Bretonneau and Guersent, have never seen stridulous laryngitis cause death. In the cases collected by English physicians, on the contrary, we see a certain number of examples of fatal termination. Whence arises this difference? always from the same cause. Many of these last authors have not been sufficiently strict in their diagnosis; whenever they have seen a paroxysm of suffocation arise rapidly in the course of apparent good health, they have admitted the existence of this disease; and it is this confounding of many diseases of very different severity which has introduced error into the results which they have obtained." (Vol. i, p. 289.)

He admits, however, on the authority of Home, Millar, Wichman, Vieusseux, and Lobstein, fatal cases, which can be referred only to stridulous laryngitis, and which M. Guersent himself regards as apparently belonging to this affection.

The best conclusion that we can come to on this subject is, that the disease described by M. Valleix and French writers generally under the name of stridulous laryngitis is a totally distinct disease from the spasmodic affection of the glottis of children known in this country and Germany by various designations, but yet in both countries regarded as a spasmodic, not an inflammatory, disease; or if inflammatory, it is considered that the inflammation does not exist in the larynx. Our main reason for coming to this conclusion is the fact that men of such experience as Bretonneau and Guersent should never have seen a case of the French disease fatal, whilst our English malady presents to every man of experience too many examples of fatality. In both diseases there is spasm of the glottis, but in the affection described by Valleix, the spasm appears to have its source in the part affected; whilst the irritation exciting the spasm in the disease of English and German writers is situated remotely from the larynx. Writers, however, are by no means agreed as to the situation of this remote irritation, and hence the multitudinous names by which the disease has been designated, such as "asthma dentientium," "asthma thymicum," &c. Indeed, we are by no means convinced that the irritation exciting the spasm is uniformly in the same locality; the variety may be in the things observed, not in the observers.

Dr. Dunglison's account of the disease, which he calls spasm of the glottis, is at once a faithful and succinct transcript of the opinions of English writers on the subject, and as such we recommend it to the student for perusal; but as it presents nothing new, we leave it without other comment.

*Croup.* The subject of croup, called by Dunglison inflammation of the larynx and trachea, and by M. Valleix pseudo-membranous laryngitis, is discussed by both writers at much length; but not with perfect accordance of sentiment. It will be observed that there is a difference in the names, that of Dr. Dunglison being comprehensive, and including all the parts especially involved in the disease, whilst that of M. Valleix is, in this respect, imperfect, but superior in another, since it depicts a characteristic of every complete case of the disease. We think, however, croup a better name than either, since it involves no disputable point either of site, nature, or tendency, and is, moreover, everywhere intelligible.

The most material difference, however, between these writers, regards the relations subsisting between croup and the diphtherite of M. Bretonneau. On this point they shall speak for themselves:

"We have seen above," says M. Valleix, "that, according to the most precise observations, it has been impossible to establish a difference of nature between pseudo-membranous pharyngeal angina and the laryngitis now under consideration. Everything, in fact, is common to them, except the seat of disease, and besides they proceed very frequently the one from the other. This is a truth now recognized, and against which the objections of certain authors, among which I shall mention those of Dr. Cheyne, (Cyclop. of Pract. Med., vol. i, p. 99,) cannot prevail. This physician does not conceive that 'we can regard these two affections as identical, because,' &c. I have cited this passage only to show how feeble are the objections urged against one of the most important results of modern observation; for, although M. Bretonneau himself has quoted a considerable number of authors who, before F. Home, had a glimpse of this truth, it must be acknowledged that it is to his able researches that we owe the complete demonstration both of the proper characteristics of diphtheritic pharyngeal angina, and of its identity with pseudo-membranous laryngeal angina. Nevertheless, as if he feared to go too far, M. Bretonneau asks (Loc. cit. p. 281) whether concretions of a non-diphtheritic nature may not be developed in the larynx. But on examining the facts attentively, we fail not to see that all the pseudo-membranes developed in the larynx are of the same nature, and that there does not exist between them any distinctive sign of material value. These two diseases differ, then, I repeat it, only in situation. But as on the one hand the difference of situation is capital, and as, on the other, diphtheritis may be limited to the larynx, the physician, whilst acknowledging that pharyngeal angina, and pseudo-membranous laryngitis are of the same nature, and are frequently confounded together, ought to make two distinct diseases of them, and study them with care separately." (Valleix, pp. 342-3.)

Dr. Dunglison takes a view very different from this. He, too, shall speak for himself. After stating the opinions of Bretonneau, and a declaration of M. H. Bell's, that the ideas of this well-known pathologist "are, at the present day, generally admitted," he says:

"It is not accurate, however, to ascribe such sentiments to the generality of pathologists. Almost all admit, that the essential character of croup, in the child, is a violent inflammation, accompanied by an exudation of plastic lymph; but the generality of American and British practitioners regard this idiopathic inflammation of the larynx and trachea, or rather of the latter—for the larynx is generally but little implicated—as very different from the diphtheritic inflammation that occurs in diphtheritic stomatitis and pharyngitis, in which, as has been shown, there is always precedent fever, and the formation of false membranes in the mouth and pharynx, which may extend down the windpipe, and give rise to symptoms of croup in the advanced stage of another and totally different disease." (Dunglison, p. 244.)

He afterwards quotes with approbation, and, we think, with success, the diagnosis of Dr. Stokes between what this writer very properly terms primary and secondary croup. The main points of his diagnosis, (independent of the difference in the locality of the primary attack,) are, that in the primary affection the fever is symptomatic of the local disease, and this fever is inflammatory; in the secondary one, prior to the occurrence of the local affection, there is a disease existing accompanied with fever, which is typhoid; again, primary croup demands antiphlogistic treatment, which is often successful; the secondary one requires the tonic, revulsive, and stimulating modes of cure; the primary disease is sporadic, never contagious, the secondary constantly epidemic and contagious (?); the first is a disease of childhood, the second affects chiefly adults; in the one the pharynx is healthy and dysphagia absent or very slight, in the other the pharynx diseased and dysphagia common and severe; in the primary disease catarrhal symptoms are often precursory to the laryngeal ones, the secondary disease supervenes without such a preliminary; in the primary disease, complication with acute pneumonia is common, in the secondary rare; lastly, in primary croup, there is no characteristic odour of breath, in secondary, the breath is often characteristically fetid.

Various circumstances mentioned in the works of our authors, and our own observation, lead us to the conclusion that Drs. Dunglison and Stokes take the correct view, that primary and idiopathic croup is a different affection from croup engrafted on diphtheritis or any other disease. One of these circumstances is the rate of mortality ascribed by authors, Caillau and Double estimating it at two thirds of those attacked; another observer, J. Frank, cured thirty-nine cases and lost twenty-seven, whilst Jurine and Vieusseux ascribe to it a fatality of one in ten only. Dr. Dunglison remarks that no such mortality attends the disease wherever he has practised and seen it; on the contrary, where the practitioner is called sufficiently early, it is usually very manageable. The scenes of Dr. Dunglison's observation have been various and extensive, comprising the banks of the Frith of Forth, the margins of the English lakes of Cumberland, London, Paris, the interior of Virginia, Baltimore, and Philadelphia. M. Valleix says that a very great discrepancy exists among authors regarding the mortality from this disease, one estimating it at almost the *totality of the cases*, another at one half, another at a ninth only. We find it impossible to reconcile these discrepancies otherwise than by supposing that certain parties have confounded in their estimates the results of the mortality of two distinct diseases, the very fatal diphtheritic laryngitis and the comparatively tractable primary croup. We would add, too, that the greater prevalence of the diphtheritic (the more fatal) species in France, is demonstrated by the very great mortality from the disease in *that*, compared with other countries. We shall venture to inquire, too, whether the very great familiarity of M. Bretonneau with diphtheritis may not arise from the nature of the locality where this gentleman practises, Tours, situated at the conflux of the Loire and Cher—whether this disease may not arise from malaria generated on the low and damp, if not marshy, banks of those rivers?

When we examine the practical precepts of our respective writers, again we see reason to conclude that they are considering different diseases. Dr. Dunglison recommends that trial should be made, at the commencement of the disease, of an emetic and a warm bath. He then says "should the symptoms not yield to the emetic and warm bath, blood must be drawn either from the arm or jugular vein, or by means of leeches;" his plan subsequently consists in the administration of large doses of the antimonial tartrate of potash; in fact, he recommends, throughout the disease, what every practitioner in this country would do in a case of croup. But what on this head says M. Valleix, or rather M. Bretonneau? "Then comes M. Bretonneau, who at first had a certain degree of confidence in bleeding, regarding it as suitable to be opposed to the progress of diphtheritis, but who was forced to abandon this view, when he saw that in no case submitted to his observation was the formation of false membrane presented by bleeding. It is in *epidemics* (who ever heard of epidemics of croup in this country?) especially that the insufficiency of bleeding has been evident to him." M. Valleix coincides in these therapeutic views of M. Bretonneau. The latter writer subsequently quotes a case in which a very abundant local bleeding was followed by a prompt extension of diphtheritis.

We consider that there is ample evidence of the non-identity of diphtheritis and croup.

We now take leave, but not *final* leave, of our authors, for we purpose at a future day to resume our critical labours on some other portions of their valuable works.

Since the foregoing observations were written, we have received a second edition of Dr. Dunglison's work,—a sufficient indication of the high character it has already attained in America,—and justly attained.

---

